# Quasi-Static Behavior of Palm-Based Elastomeric Polyurethane: For Strengthening Application of Structures under Impulsive Loadings

**DOI:** 10.3390/polym8050202

**Published:** 2016-05-20

**Authors:** H. M. Chandima Chathuranga Somarathna, Sudharshan N. Raman, Khairiah Haji Badri, Azrul A. Mutalib, Damith Mohotti, Sri Devi Ravana

**Affiliations:** 1 Department of Civil and Structural Engineering, Universiti Kebangsaan Malaysia, 43600 UKM Bangi, Malaysia; hmccsomarathna@gmail.com (H.M.C.C.S.); azrulaam@ukm.edu.my (A.A.M.); 2 Department of Architecture, Universiti Kebangsaan Malaysia, 43600 UKM Bangi, Malaysia; 3 School of Chemical Sciences and Food Technology, Universiti Kebangsaan Malaysia, 43600 UKM Bangi, Malaysia; kaybadri@ukm.edu.my; 4 School of Civil Engineering, The University of Sydney, 2006 New South Wales, Australia; damith.mohotti@sydney.edu.au; 5 Department of Information Systems, University of Malaya, 50603 Kuala Lumpur, Malaysia; sdevi@um.edu.my

**Keywords:** palm-based polyurethane, elastomer, impulsive loadings, quasi-static, retrofitting, strengthening

## Abstract

In recent years, attention has been focused on elastomeric polymers as a potential retrofitting material considering their capability in contributing towards the impact resistance of various structural elements. A comprehensive understanding of the behavior and the morphology of this material are essential to propose an effective and feasible alternative to existing structural strengthening and retrofitting materials. This article presents the findings obtained from a series of experimental investigations to characterize the physical, mechanical, chemical and thermal behavior of eight types of palm-based polyurethane (PU) elastomers, which were synthesized from the reaction between palm kernel oil-based monoester polyol (PKO-p) and 4,4-diphenylmethane diisocyanate (MDI) with polyethylene glycol (PEG) as the plasticizer via pre-polymerization. Fourier transform infrared (FT-IR) spectroscopy analysis was conducted to examine the functional groups in PU systems. Mechanical and physical behavior was studied with focus on elongation, stresses, modulus, energy absorption and dissipation, and load dispersion capacities by conducting hardness, tensile, flexural, Izod impact, and differential scanning calorimetry tests. Experimental results suggest that the palm-based PU has positive effects as a strengthening and retrofitting material against dynamic impulsive loadings both in terms of energy absorption and dissipation, and load dispersion. In addition, among all PUs with different plasticizer contents, PU2 to PU8 (which contain 2% to 8% (*w*/*w*) PEG with respect to PKO-p content) show the best correlation with mechanical response under quasi-static conditions focusing on energy absorption and dissipation and load dispersion characteristics.

## 1. Introduction

In recent years, substantial efforts by various researchers have been assessed to identify novel and cost-effective solutions, and their feasibility to minimize damage to buildings and infrastructures caused by terrorist activities and accidental explosions [1,2,3,4,5,6,7]. The use of elastomeric polymers to strengthen and retrofit applications has attracted attention because elastomeric polymers may be able to absorb the energy generated by dynamic and impulsive blast and ballistic loading. An initial attempt to utilize elastomeric polymers for such purpose was undertaken by the Air Force Research Laboratory at Tyndall Air Force Base, Florida, to evaluate the applicability of 21 types of commercially available polymers, including seven extruded thermoplastic sheet materials, 13 spray-on materials (seven polyurethane, one polyurea, and five polyurea/urethane systems), and one brush-on material in enhancing the structural performance of masonry and lightweight steel structures under blast effects [8]. These initial investigations deduced that elastomeric polymer enhances the resistivity of structures under such loading environment and reduces fragmentation and crack propagation [8]. Several studies demonstrate the potential of elastomeric polymers as retrofitting and strengthening material in several types of structural elements, on masonry structures [8,9,10,11,12,13], metallic structures [14,15,16,17,18,19,20,21,22,23,24,25,26,27,28], composite structural systems [29,30,31,32,33,34,35,36], and reinforced concrete structures [37,38,39,40]. Even though reinforced concrete is the most widely used construction material worldwide, research and application of this technique on reinforced concrete structures is limited. This research attempts to investigate the suitability of eight types of polyurethanes (PUs) as a structural strengthening material for coating application to enhance the resistivity of reinforced concrete structures against blast effect. A comprehensive understanding of the physical, mechanical, thermal, and chemical properties, including their behavior, is essential to propose these materials as an effective and feasible alternative to existing structural retrofitting materials.

PU is considered a versatile material because of its outstanding characteristics and morphology, and its ability to alter its microstructure leads to a wide range of mechanical behaviors [41,42,43,44,45,46,47]. Compared with rubber and other elastomers, PU has numerous benefits, such as higher load bearing capacity, cut and tear resistance, pourability (castability), a wide durometer range, microorganism resistance, oil and petroleum resistance, low or high rebound, and versatility [47]. Urethane link is formed by a rapid chemical reaction between isocyanate (–N=C=O groups) and hydroxyl groups (–OH). Furthermore, PU can be designed to have any properties to fulfil material requirements for its application by altering the chemical composition [42,48,49]. Macro-diol, diisocyanate, and plasticizer are the three main chemical components of PU; generally, the nature of polyol chain, urethane group concentration, and other functional groups in the PU structure has a profound effect on physical and mechanical properties [42]. The mechanical properties of PUs are significantly interrelated with the proportion of the OH and NCO in the PU networks, and are directly correlated with the hydroxyl number of the polyol and thus with the concentration of the urethane group in PU networks [50]. The intermolecular forces in PU networks play a significant role in the properties of PU. PU is a linear segmented blocked copolymer that contains soft and hard segments from OH and NCO groups, respectively. The rubbery behavior of PU is mainly due to the low glass transition temperature of soft segments, and the high glass transition temperature of hard segments provide PU with glassy and/or crystalline properties. Microseparation of these domains due to the dissimilarity of these segments’ properties is responsible for the wide range of properties of PU, including a majority of other elastomeric polymers [51]. Several researchers studied the mechanical behavior of PU elastomer under quasi-static conditions [52,53,54,55,56,57,58,59,60]. Sarva *et al*. [61] and Russo and Thomas [62] demonstrated that an increase in hard segments results in high ultimate strength and initial modulus, as well as a low elongation at rupture. O’Sickely *et al*. [63] evaluated the hard segment’s effect on PU’s mechanical properties under a narrow range of hard segments and concluded that it has little influence on the properties and morphology.

Strength and elongation characteristics, cost effectiveness, easy application, and the problems associated with the environment and flammability should be considered when selecting an appropriate polymer for blast strengthening applications [8]. In Knox *et al*.’s [8] initial investigation, polyurea was selected among 13 commercially available spray-on polymers for further evaluation as a retrofitting material because of its strength, cost, and flammability. Because in their research, polyurea was typically stiffer than PU, and it elongated to a lesser extent compared with the PU. However, Bahei-El-Din and Dvorak [29] showed that the behavior of the sandwich plates with hyper-elastic PU shows slightly better performance under energy absorption compared with the design that contains rate-dependent, elastic-plastic polyurea interlayer, with nearly similar other benefits. Furthermore, under static loads, PU interlayer has less deflection in the sandwich plates compared with polyurea. Table 1 tabulates the quasi-static mechanical and physical properties of selected elastomeric polymers (PU and polyurea) used as strengthening material for blast and ballistic loads as reported by various researchers [9,19,25,29,30,33,35,36,37,51,64]. This paper addresses the analysis from a series of experimental investigations to characterize the physical, mechanical, chemical and thermal behavior of palm-based PU elastomers, which were synthesized as coatings for structural strengthening application in enhancing the impulsive resistance of reinforced concrete structures.

## 2. Materials and Methods

### 2.1. Materials

Palm-based polyol (PKO-p) (molecular mass ≈ 477 g/mol, hydroxyl value of 350–370 mg KOH/g, and moisture content of 0.09%, viscosity of 374 cps, and specific gravity of 0.992 g/cm^3^ at ambient temperature) [65,66] was supplied by the Polymer Research Centre (PORCE) of Universiti Kebangsaan Malaysia (Bangi, Malaysia). A more detailed description on the synthesis of the (PKO-p) is provided in Badri [65] and Badri *et al*. [66]. Meanwhile, the 4,4-diphenylmethane diisocyanate (MDI) was obtained from Cosmopolyurethane Sdn. Bhd., Kuala Lumpur, Malaysia. Acetone (industrial grade) and polyethylene glycol (PEG: *M*_w_ 200 Da) were purchased from Sigma Aldrich (M) Sdn. Bhd., Petaling Jaya, Malaysia.

### 2.2. Synthesis of the Palm-Based PU Elastomer

The palm-based PU elastomer was synthesized from the rapid reaction of PKO-p and MDI via pre-polymerization under ambient temperature without any catalyst. When a structure is subjected to highly impulsive loads, it undergoes a severe loading accompanied by high strain conditions. In view of this, a strengthening material should have both high elongation capacity and modulus characteristics. With the aim of enhancing its elongation capacity, based on the findings of initial investigations, PEG was added as a plasticizer and eight types of elastomeric PUs were synthesized in this study by varying the PEG content over a narrow range from 0%–15% *w*/*w* with respect to the weight of the PKO-p. The dominantly contributing OH group is the polyol, and a short chain plasticizer (PEG 200) was used in order to avoid chain entanglement and to maintain the required stiffness since the higher molecular plasticizers may lose the stiffness properties rapidly. The mix proportion of the PKO-p and MDI (100:80) was kept constant throughout the experiment. MDI and PEG were added according to the PKO-p weight, and acetone was added based on the total weight of –OH system and –NCO system separately with the same percentage of 35% *w*/*w* (–OH system contains the mix of PKO-p and PEG, and –NCO system contains MDI).

Eight types of PUs were synthesized using the solution casting process; these PUs are PU0, PU2, PU4, PU6, PU8, PU10, PU12, and PU15 (PU6 indicates the PU that contained 6% *w*/*w* of PEG). Clear yellowish and bubble-free precast PU elastomeric sheets were obtained and left to condition at ambient temperature for further characterization.

### 2.3. Fourier Transform Infrared (FT-IR) Spectroscopy Analysis

FT-IR spectra of the PU elastomer were analyzed to identify the functional group of each PU sample by using an FT-IR spectrophotometer (model Perkin Elmer Spectrum 400 FT-IR, PerkinElmer, Inc., Waltham, MA, USA) with the attenuated total reflectance (ATR) technique at a wave number that ranges from 4000 to 500 cm^−1^.

### 2.4. Density Determination

The densities of the eight types of PUs were evaluated following the mass over volume method. The samples were cut and cleaned to remove surface debris, and the test was conducted in ambient temperature. Dimensions of each specimen were measured by using a vernier caliper with an accuracy of 0.01 mm, and the average of the three values was used for the calculations. Mass was measured by using an electronic balance with a milligram accuracy (0.001 g). The average density values were determined through ten measurements for each type of PU.

### 2.5. Shore D Hardness Test

Shore D hardness test was performed on an analog Shore D durometer hardness tester (Cole-Parmer Instrument Co Ltd., London, United Kingdom) according to the ASTM D2240, and test specimens whose length of each side is larger than 30 mm were used. Two 3 mm thick PU sheets were placed as a bundle. The accuracy of the durometer hardness tester was checked by using the reference material (provided by the supplier) before the test was performed. The average value of the five determinations of hardness at different positions on the specimen was calculated as the hardness value of the PU sample.

### 2.6. Tensile Test

**Figure 1:**
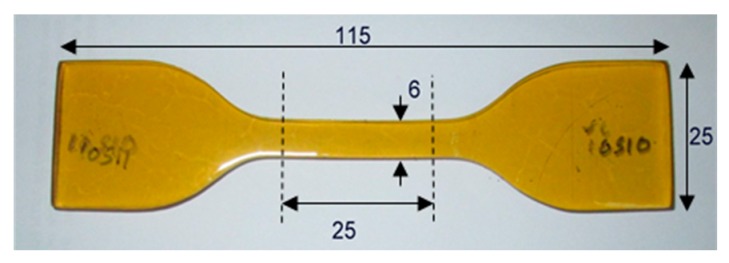
Sketch of the tensile test specimen with dimensions (Note: Not to scale, all dimensions shown are in mm).

The uniaxial tension test was performed on an Instron Universal Testing Machine (Instron Corporation, Canton, MA, USA), Model No. 5566 under quasi-static condition with displacement-controlled condition in accordance with ASTM D412. Dumbbell test specimens (3 mm thick) were obtained from precast PU sheets in the same direction of the sheet to minimize the influence of anisotropy or grain directionality caused by the flow’s direction during preparation and processing. All dumbbell test specimens (Figure 1) were cut using Die C in accordance with the procedure described in the ASTM specifications. The dimensions were measured by using a vernier caliper with an accuracy of 0.01 mm, including the average of three measurements used for the dimensions of each specimen.

**Figure 2:**
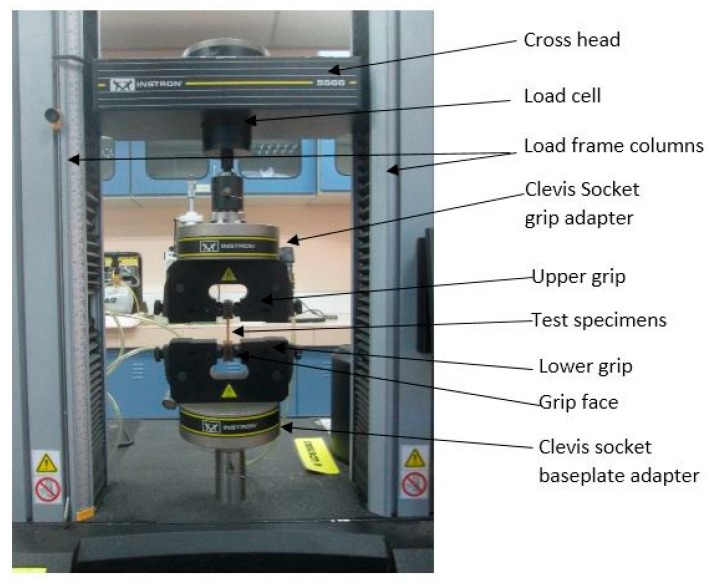
Tensile test setup.

All test specimens were automatically clamped into the grip. A uniform rate of 50 mm/min was used for grip separation during the test (Figure 2). All test specimens were tested at ambient temperature, and data were measured using Blue Hill v2.5 software (Instron Corporation, Canton, MA, USA). The time, load, and extension data were recorded up to failure, and tensile characteristics were calculated by using obtained data.

### 2.7. Flexural Test

**Figure 3:**
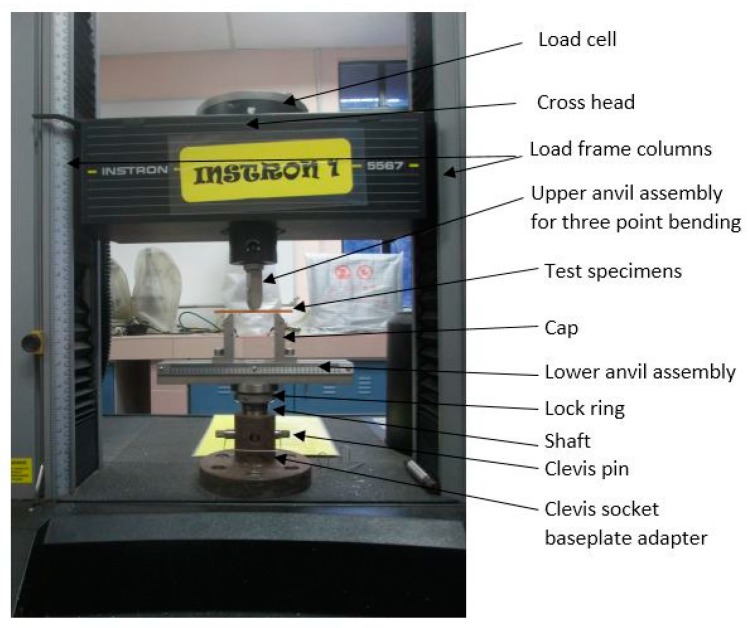
Flexural test setup.

Flexural test was conducted according to ASTM D790 utilizing three-point bending test with Instron Universal Testing Machine (Instron Corporation, Canton, MA, USA), Model No. 5567 (Figure 3). All test specimens were cut in accordance with the procedure specified, with 3 and 15 mm as the average depth and width, respectively, and 48 mm as the effective support span. The total length of the specimens was 110 mm, thereby allowing sufficient overhang on each end to avoid slipping through the supports.

All test specimens were tested at ambient temperature, with a cross-head motion of 12.8 mm/min to obtain 0.1 min^−1^ strain condition, considering that failure was not observed within 0.05 strain under 0.01 min^−1^ strain condition. The test was continued until the strain exceeded 0.1. Time, load, and deflection were measured using Blue Hill v2.5 software (Instron Corporation, Canton, MA, USA). The average of three measurements was used for each dimension. The modulus of elasticity, flexural stress, and strain at maximum stress were obtained as the average of five readings.

### 2.8. Izod Impact Test

Numerous methods are used to evaluate the resistance capacity and the behavior against impact loading conditions of elastic and plastic polymer materials, such as Izod, Charpy, tensile impact, and Gardner tests. The Izod pendulum test method is one of the most extensively used techniques by most researchers and industries, and tests are detailed in several standards (ASTM D256-05, ISO 180:2000) [67,68]. To perform the Izod impact test, rectangular specimens were cut from the 3-mm-thick precast PU sheets with a width of 13 mm and a length of 64 mm. During the preparation of specimens, special attention was given to maintain the faces of the flat and parallel specimens, considering that they may be highly sensitive to clamping pressure [67]. Generally, notches produce stress concentration and increase the possibility of brittle failure rather than ductile failure. Polymeric materials, such as PU, perform poorly in the notched Izod impact test because these materials are sensitive to stress concentrations at the notch; furthermore, crack propagation is initiated in the samples [67,69]. To overcome this problem, un-notched specimens were used in the test. During the positioning of the specimens, each specimen was examined to ensure that it was free from twisting, scratches, sink marks, and pits. Specimens were positioned in a line 22.00 mm above the top surface of the specimen holder and at the center of the striker to reduce the vibration of the pendulum arm. Elastomeric materials are extremely sensitive to clamping pressure. Accordingly, the specimens were clamped into the grips with a roughly equal minimum pressure to prevent movement during impact without allowing any pressure deformation. The impact tests were conducted with a pendulum speed of approximately 3.46 m/s at the moment of impact. Considering environmental factors, such as temperature and atmospheric moisture further playing a significant role in impact resistance [67,68], the test was conducted at a constant ambient temperature and conditions. The lost energy, which was needed to break the PU samples, is the energy lost per unit cross-sectional area at the break point in units of J/m² calculated based on the changes in the distance of pendulum following through on its path.

### 2.9. Differential Scanning Calorimetry (DSC) Analysis

DSC analysis was performed on a Mettler-Toledo (model DSC 822e, Mettler-Toledo, Greifensee, Switzerland). A 4-mg sample was scaled in aluminum pans with a perforated lid. The heating rate was fixed to 10 °C/min with a nitrogen flow of 20 mL/min. The test was performed in a temperature range from 25 to 250 °C, and the glass transition point (*T*_g_) was determined.

## 3. Results and Discussion

The results of the experimental studies and their discussions are provided in this section. Clear yellowish and bubble-free PU elastomer was obtained after curing. The curing time increased with the percentage of the PEG content. These investigations allow the analysis of the behavior under controlled laboratory conditions; which subsequently, are used to select materials for the required applications and for quality control during the operation.

### 3.1. FT-IR Analysis

**Figure 4:**
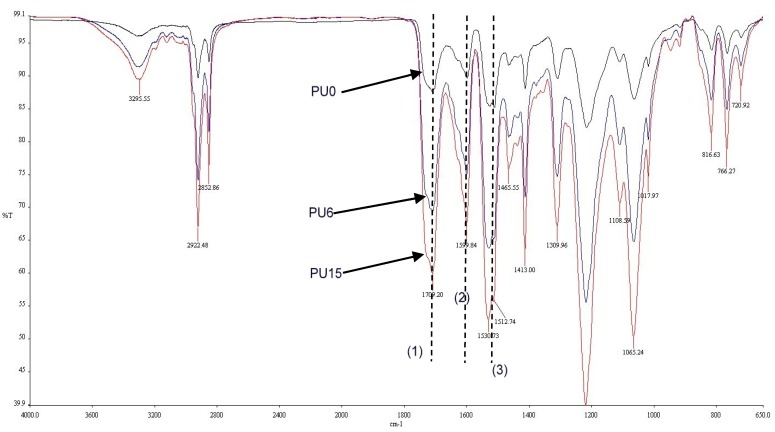
FT-IR spectrums of the PUs (PU0, PU6 and PU15 are types of PU and PU6—PU which contained 6% PEG).

FT-IR analysis was conducted to examine the functional groups in PU systems. Figure 4 shows the FT-IR spectrums obtained for PU0, PU6, and PU15. The main functional groups in PU, which are C=O, C=C, and C–C, are indicated using reference numbers 1, 2, and 3, respectively, in Figure 4.

### 3.2. Density

Average density values from PU0 to PU15 showed only a small deviation from one another, and the average density is 1075 kg/m^3^ for all types of PUs. Thus, the influence of the content PEG on the density is negligible. The reported average densities of elastomeric polymers (PU and polyurea) used by previous research for retrofitting against impulsive loadings range from 950 to 1200 kg/m^3^. Yi *et al*. [51] reported the density of three different PU types as 1140, 1128, and 1113 kg/m^3^. Bahei-El-Din and Dvorak [29] used the average density of PU as 1200 kg/m^3^, while Grujicic *et al.* [35] used 1104 kg/m^3^ for blast strengthening in a sandwich composite system in their numerical studies. Therefore, the density of palm-based PU is within the allowable range, compared with the results obtained by other researchers. 

### 3.3. Shore D Hardness Test

**Figure 5:**
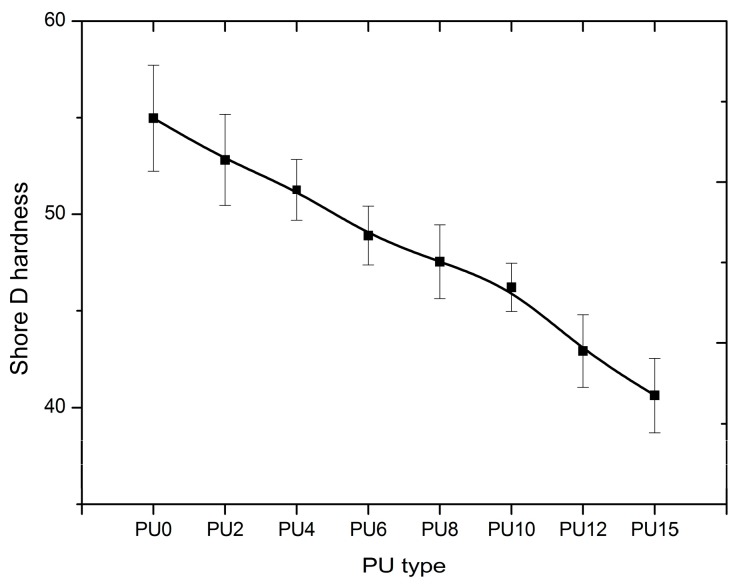
Shore D-Hardness value (PU0–PU15 are types of PU and PU6—PU which contained 6% PEG).

Hardness depicts the ability of a material to resist plastic deformation by penetration, resist bending, abrasion, scratching, or cutting. This test is analyzed based on the penetration depth created by a specific type of indentor when penetrating the material under specific forces and conditions. The indentation hardness value is inversely related to the penetration depth and it is correlated to the elastic modulus and elastic behavior of the material. Figure 5 plots and compares the Shore D hardness values. Results show the highest hardness value for PU0, which gradually decreased from PU0 to PU15. The Shore D hardness values of all types of PUs lie in the range from 40 to 56, which is approximately the typical hardness value of a skateboard wheel [70]. As stated in the Shore D scale, all types of PU can be categorized as hard to extra-hard polymers [70].

Shore D hardness value of 63 ± 3 was observed by Mohotti *et al*. [25] for their polyurea sample that was used to coat composite aluminum plates, which were subjected to high impact loadings. Hardness and ductility are essential characteristics for a material to be used for strengthening applications, such as protective coating for structures subjected to impulsive loadings.

### 3.4. Tensile Test

#### 3.4.1. Tensile Characteristics

**Figure 6:**
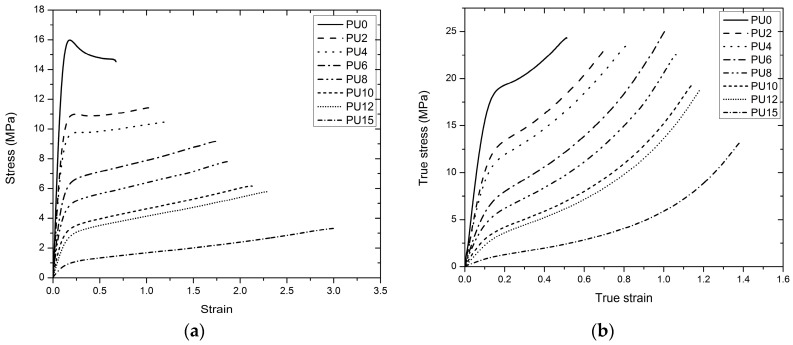
Tensile stress–strain behavior (**a**) engineering, (**b**) true, of the all types of PU, (PU0–PU15 are types of PU and PU6—PU which contained 6% PEG).

**Figure 7:**
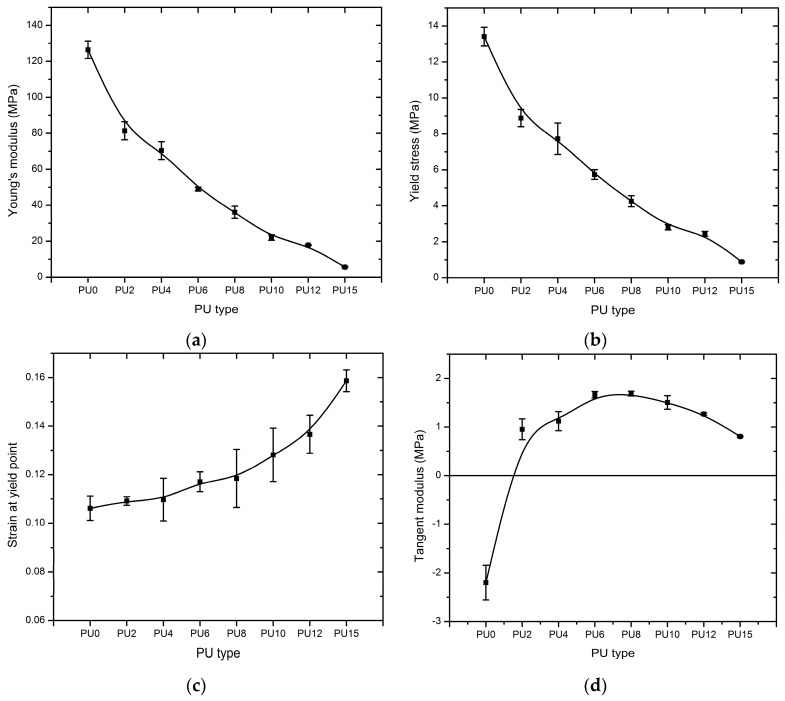
Comparison of tensile properties of the PUs, (**a**) Young’s modulus; (**b**) yield stress; (**c**) strain at yield point; (**d**) tangent modulus. (PU0–PU15 are types of PU and PU6—PU which contained 6% PEG).

The tensile stress responses (Engineering and True) of the eight types of PUs that correspond to the strain profiles are plotted on the graph of Figure 6a,b. Engineering stress-strain is used for the analysis. All stress-strain curves follow the typical behavior of an elastic-plastic material. These curves exhibit an initial linear region. Following this linear region, the PUs initiated yielding after reaching significant stress and elongation. As shown in Figure 7a, Young’s modulus was the highest for PU0 and rapidly decreased from PU0 to PU2 with the addition of plasticizer. Subsequently, it decreased gradually until PU15 with the increasing plasticizer content and reduced by 36%, 44%, 61%, 71%, 83%, 86%, and 96% compared with PU0. These results verify the behavior obtained by Tsou *et al.* [71]. 

The addition of plasticizer contributed to the reduction of the modulus considering that the increase in the length of polymer chain leads to a high mobility in the molecular structure. Young’s modulus values of PUs (except PU15) are within the range of Young’s modulus values of elastomers used by other researchers as strengthening material in these applications (Table 1). The stress–strain relationship indicated that all types of PUs deform significantly after reaching the yield point and will not fracture suddenly without warning prior to failure. PU15 exhibits higher performance and PU0 exhibits lower performance under this behavior. The variations of the yield stress and strain at the yield point are depicted in Figure 7b,c, respectively. The yield stress decreased from PU0 to PU15, whereas the yield strain increased within a narrow range. Raman *et al.* [37] reported a yield stress of 5.5 MPa in for the polyurea sample that they studied, which was subsequently used to strengthen the reinforced concrete structures against blast loadings. PU0 to PU6 samples synthesized in the present study exhibited yield stresses that were higher than the value reported by Raman *et al*. [37]. 

Furthermore, the variation of the yield stress is similar to the trend of the variation of the Young’s modulus, exhibiting a rapid reduction of yield stress with the addition of plasticizer from PU0 to PU2 and subsequently experiencing a gradual decrease. The PUs underwent a brief yielding, where the interbonding within the molecular structure was broken down. Generally, at strains beyond the yield point, the breakdown of the two-phase structure (hard segments and soft segments) is initiated due to the breakdown of cross-linkages. This results in sliding of hard segments relative to their adjacent segments within the hard domains, breaking the original hard domains into several smaller units and stripping of segments from the hard domains and formation of new soft matrix within the hard domains. This subsequently results in irreversible deformations and residual strain in the material [72,73].

Subsequently, further application of the tensile load resulted in a slight increase in the stress value except in PU0; PU2 to PU15 were subjected to a strain hardening mechanism, whereas PU0 was subjected to a strain softening mechanism. The tangent modulus of all PUs is presented in Figure 7d and is a negative value for PU0 because of the strain softening mechanism. A positive value was given for others because of the strain hardening mechanism, which resulted from elasticity caused by the addition of plasticizer. Within this region, PUs exhibited an almost linear shape in the curves prior to subsequent failure after considerable elongations.

**Figure 8:**
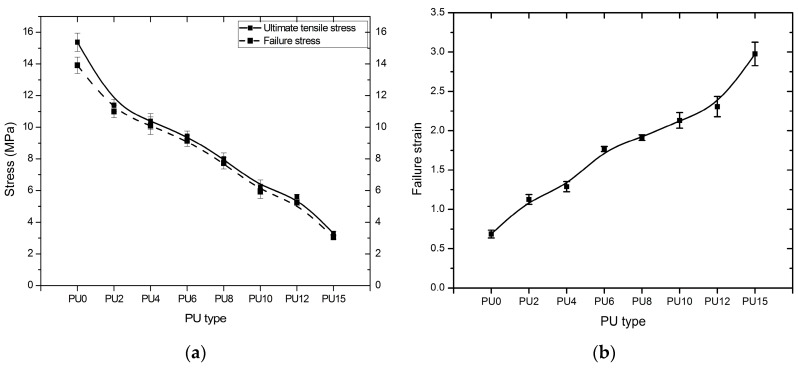
(**a**) Ultimate and failure tensile stress, (**b**) failure strain, of the PUs (PU0–PU15 are types of PU and PU6—PU which contained 6% PEG).

PU0 reached its ultimate tensile stress value after its yield point, and other PUs reached their ultimate tensile stress slightly before failure. Ultimate tensile stress and failure stress values are shown in Figure 8a. As shown in Figure 8a, except for PU0, other PUs demonstrate almost equal failure stress compared with their ultimate tensile stress, considering that they reached their ultimate tensile stress slightly before their failure. The variation of both ultimate tensile stress and failure stress show a similar trend to the Young’s modulus and yield stress. The failure occurred at 13.91, 11.00, 10.10, 9.15, 7.73, 5.96, 5.25, and 3.04 MPa stresses in PU0, PU2, PU4, PU6, PU8, PU10, PU12, and PU15, respectively, at 0.69, 1.13, 1.29, 1.77, 1.91, 2.13, 2.31, and 2.98 strains. Figure 8b shows a comparison of the PU elastomers’ failure strain. A clear increase in the failure strain with the increasing plasticizer content was observed, and the same behavior was observed by Tsou *et al*. [71] and Delpech and Coutinho [74]. Except PU0, all other PU types exhibit more than 100% elongation capacity. Furthermore, PU15, which contains the maximum plasticizer content (15%), shows the highest strain capacity of nearly 3.0. Short chain segments provide high resistance against mechanical properties. Increasing the length of the soft segment enhances the elastomeric characteristic of polymers. Another imperative characteristic of elastomeric polymers is a high elongation capacity, which can be more than 1.00. A high elongation capacity assists in obtaining a high-energy absorption capacity. Furthermore, it acts as an essential factor to reduce impulsive effect and fragmentation during detonation. In previous studies, researchers used elastomeric polymers with tensile strain that ranges from 0.89–3.50 (Table 1). Except for the tensile strain of PU0, the remaining seven types of PU exhibit tensile strain within that range.

**Figure 9:**
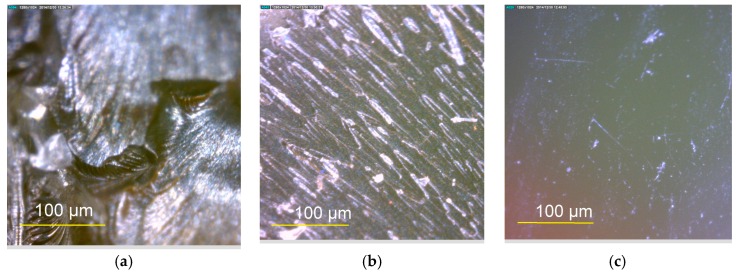
Failure surfaces of PU samples of tensile test. (**a**) PU0; (**b**) PU6; (**c**) PU15.

Generally, all the stresses seemed to be reduced, and failure strain increased from PU0 to PU15 as a result of the increment of the plasticizer content. Furthermore, 15% plasticizer resulted in 80%–90% reduction in the stresses and a 432% increase in the failure strain compared with the control (PU0). This result verifies the role of the plasticizer, considering that it increases the chain length of the polymer because of random polymerization that creates two different end groups—one that belongs to the plasticizer and the other to the palm-based polyol—and results in enhanced elasticity. The tensile test indicates that the addition of plasticizer increases the elastic and ductile characteristics, thereby decreasing the stresses and Young’s modulus, whereas the tensile strain at failure increased. The failure surfaces of the tensile test specimens were examined using a Dino-Lite optical microscope with a magnification of 500 times (Figure 9). The failure of PU0 was brittle whereas PU15 showed a smooth ductile failure. The ductility of the failure surface increased from PU0 to PU15, displaying an intermediate failure pattern in PU6 unlike in PU0 and PU15. The overall findings indicate that the stiffness of PU changed significantly, and the behavior changed from leathery to rubbery with increasing plasticizer content. PU0 shows the behavior of a material that has high stiffness but low toughness. The behavior of PU15 was similar to that of a material that has high toughness but low stiffness. PU2, PU4, PU6, and PU8 exhibited the behavior of a material with desirable stiffness and toughness qualities, which are acceptable as a strengthening material for blast retrofitting under tensile characteristics.

#### 3.4.2. Strain Energy

Even though several methods are used to measure the energy absorption and dissipation capacities of materials, strain energy is a vital characteristic when measuring the energy absorption and dissipation capacities of any elastomeric material. In the stress–strain curves of the PUs, the initial linear region or the deformation caused by the axial load is unaccompanied by any energy dissipation. The application of axial load on the PU is stored as strain energy throughout its volume, thereby resulting in elastic deformation [75]. The modulus of resilience (*U*_r_) shows the capability of a material to absorb energy when it is deformed elastically, and energy is dissipated during the unloading of the applied loads. Specifically, it can be defined as the ultimate energy that can be absorbed per unit volume in the elastic region without undergoing permanent damage due to deformation. The density of energy was calculated by taking the area under the stress–strain curve until the yield limit was reached. 

**Figure 10:**
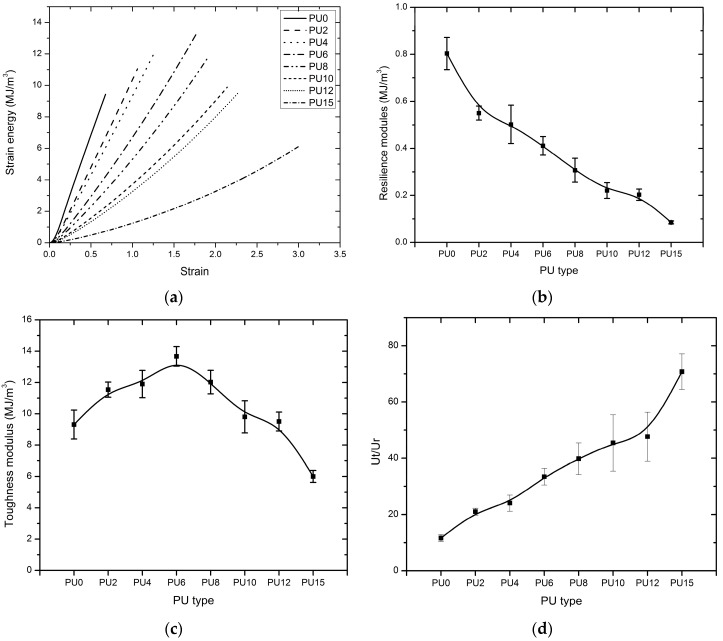
Comparison of strain energy of the PUs (PU0-PU15 are types of PU and PU6—PU which contain 6% PEG), (**a**) total strain energy *vs.* strain; (**b**) resilience modulus (*U*_r_); (**c**) toughness modulus (*U*_t_), (**d**) toughness to resilience modulus ratio (*U*_t_/*U*_r_).

On the other hand, toughness is the ability of a material to absorb energy with elastic and plastic deformation without fracturing; the modulus of toughness (*U*_t_) is the density of strain energy of the material before experiencing failure [75]. It can be defined as the work performed on a unit volume of PU material under an axial load until the failure. This modulus was calculated by taking the entire area under the stress–strain curve from the origin to failure. Figure 10a shows the cumulative strain energy *vs.* strain, whereas the variations of modulus of resilience, *U*_r_, and the modulus of toughness, *U*_t_, with the types of PU are presented in Figure 10b,c. As illustrated by the graphs, the resilience modulus decreased steadily from PU0 to PU15; while the toughness modulus increased gradually up to PU6, with a subsequent gradual reduction up to PU15. Resilience modulus values of PU0 to PU6 were higher than the value reported by Raman *et al*. [37] for the polyurea sample that was used in their study to strengthen the reinforced concrete structures against blast loadings. Apparently, the toughness modulus of the PUs was considerably higher than that of the counterpart in the elastic region. Figure 10d depicts the ratio between the toughness and resilience moduli of the PU materials evaluated in this study, which were nearly ten to seventy times tougher than their resilience modulus. These findings methodically demonstrate that the PUs can absorb a considerable amount of energy even if they undergo plastic deformation. This characteristic is important for a material that will be used for strengthening applications. This finding is consistent with the objectives of this study, that is, to develop a material for strengthening structures subjected to impulsive loading conditions. The ability to absorb a considerable amount of energy is preferred in a strengthening material, unlike most brittle construction materials, such as masonry, concrete and ceramics, which would fail abruptly after yielding.

### 3.5. Flexural Test

**Figure 11:**
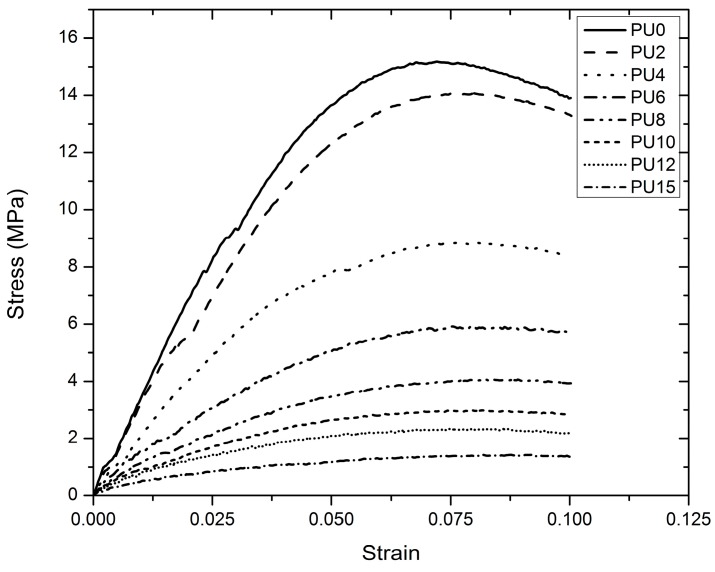
Flexural stress–strain behavior of the all types of PU, (PU0–PU10 are types of PU and PU6—PU which contained 6% PEG).

**Figure 12:**
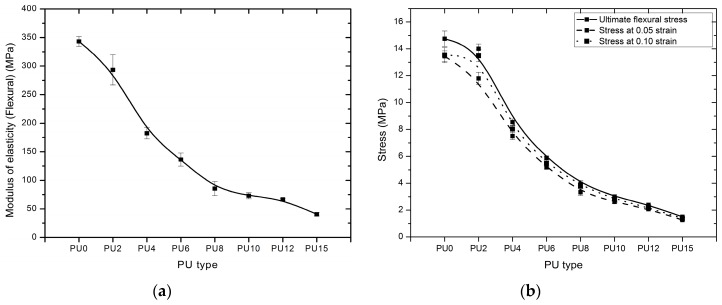
Comparison of (**a**) modulus of elasticity (flexural); (**b**) stresses at 0.05 and 0.10 strains of the PUs (PU0–PU10 are types of PU and PU6—PU which contained 6% PEG).

Flexural test was conducted to investigate the load dispersion ability of PU elastomers. The modulus of elasticity (flexural), ultimate flexural stress, and stresses at 0.05 and 0.10 strain conditions were obtained and compared. Figure 11 depicts the stress–strain curves for each PU type, and failure did not occur for all types of PUs. The test was continued until the strain exceeded 0.1, and a comparison was conducted until the behavior reached 0.1 strain conditions. PU0 exhibited the highest modulus of elasticity; this modulus decreased from PU0 to PU15 with increasing plasticizer content, as shown in Figure 12a. A comparison between ultimate flexural stress and stresses at 0.05 and 0.10 strains is shown in Figure 12b. PU0 has the highest flexural stress values for all cases, and all the stresses reduced rapidly from PU0 to PU4. Subsequently, they decreased gradually from PU4 to PU15, and the lowest stress values were observed for PU15. The flexural stress values decreased with the addition of plasticizer. The ultimate flexural stress and stresses at 0.05 and 0.10 strains were reduced by nearly 90% with 15% plasticizer (PU15). Even though the ultimate flexural stress was reduced with increasing plasticizer content, the strain at ultimate flexural stress shows a nearly equivalent strain (~7.5%–8%) for all types of PUs.

### 3.6. Izod Impact Test

Under high strain rate conditions, such as high impulsive blast and ballistic loadings, polymers can exhibit brittle rather than ductile failure. Therefore, structural components could fail catastrophically at a lower load condition than expected [61,62,67]. Understanding the behavior of polymers as a function of impact rate can provide guidance on how polymers should be used in these strengthening applications. Impact test is an experimental method to quantify the ability of energy absorption and dissipation by polymers under extreme loading conditions [67,69]. Impact behavior is a key factor in the toughness of materials [67,68].

Total impact energy is the energy lost by the pendulum during the breaking of the specimen and is the total required energy to initiate fracture of the PU specimen, propagate the fracture through the PU specimen, cause plastic deformation of the specimen at the fracture line, throw the free end of the broken specimen, and bend the PU specimen. The following assumption was made: Negligible energy is required to generate vibration in the pendulum arm, produce movement of the frame test apparatus or base, overcome the forces produced by friction and windage in the pendulum bearing, and overcome the friction forces produced by the rubbing of the striker over the bent specimen. 

**Figure 13:**
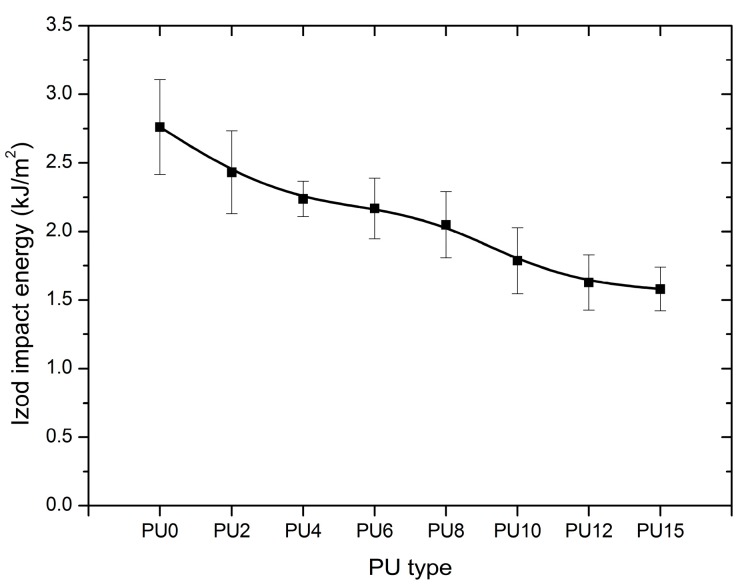
Impact energy, (PU0–PU15 are types of PU, and PU6—PU which contained 6% PEG).

Complete break was observed during the impact test for all types of PUs. Figure 13 shows the absorbed impact energy with respect to the PU type, and the trend shows a gradual reduction of the impact energy from PU0 to PU15. As shown in Figure 13, the use of plasticizer reduced the impact energy by 12%, 19%, 22%, 26%, 35%, 41%, and 43% for PU2, PU4, PU6, PU8, PU10, PU12, and PU15, respectively, with reference to the control (PU0). With increasing PEG content, PU elastomer functioned as a plasticizer in the composite system, considering that it increases the length of the polymer chain while increasing the movement ability. Even though the addition of plasticizer resulted in a noticeable decrease of absorbed impact energy, it increased the elongation capacity significantly. This improvement may be attributed to efficient energy absorption and dissipation through elastic and plastic deformations, as observed in uniaxial tensile tests.

### 3.7. DSC Analysis

DSC analysis displays heat effects accompanied by chemical reactions and phase transitions as a function of temperature. The function of temperature was obtained using the difference in heat flow to the PU sample and a reference (empty aluminum pan) at the same temperature while increasing the temperature of both sample and the reference with constant rate (10 °C/min). Considering that the DSC analysis was conducted at constant pressure, heat flow is shown, and it is equivalent to the change in enthalpy.

(1) $$\left(\frac{dq}{dt}\right)p=\left(\frac{dH}{dt}\right)$$

where d*H*/d*t* indicates the heat flow, and the difference of the heat flow between the PU sample and the empty aluminum pan is expressed as follows:

(2) $$\Delta \left(\frac{dH}{dt}\right)=\left(\frac{dH}{dt}\right)\text{PU sample }-\;\left(\frac{dH}{dt}\right)\text{empty aluminum pan}$$

Considering that this process is exothermic, similar to most polymer reactions, heat is dissipated. Consequently, Δ(d*H*/d*t*) has a negative value because heat flow to the PU sample is lower than that of the empty aluminum pan.

Glass transition temperature (*T*_g_), which is the temperature at which PU polymers are transformed from a brittle, glassy state to a rubbery state, for all PU types was obtained. *T*_g_ decreased considerably with the addition of plasticizer into the PU matrix system. Table 2 tabulates the values. PEG 200 was used as the plasticizer, which embedded between the PU chains while increasing the spacing and free volume in between. Added PEG allows polymer chains to move one another even at lower temperatures and results in decreased stiffness of PU elastomers when the *T*_g_ has been reached. Grujicic *et al.* [36] showed that the mechanical response of elastomeric polymer under impact conditions is sensitive to the difference between the *T*_g_ of the elastomeric polymer and the reference test temperature. When this difference is large, the polymer tends to exhibit the high ductility behavior of conventional elastomers in their rubbery state. When the test temperature and *T*_g_ are closer, glassy behavior is observed during deformation. The transition process is associated with viscous energy dissipation. This mechanism provides additional energy absorbing and dissipating capabilities, and may further contribute to superior protection ability of elastomeric polymers against blast and ballistic conditions.

## 5. Conclusions

Selected chemical, physical, mechanical and thermal properties of eight types of PUs, which have different plasticizer contents, were discussed in this study. The findings are compared with the results obtained by other researchers. Shore D hardness tests showed a higher hardness value relative to other types of elastomeric polymers, and all types of PUs can be categorized as hard to extra hard polymers. The stress–strain relationship shows that all PU materials follow the typical behavior of an elastic-plastic material. These deformations (brief yielding) occur even after the ultimate elastic limit was reached, and these materials will not fracture suddenly without warning prior to failure. PU15 exhibits the best performance, whereas PU0 shows the poorest performance among all PUs under this behavior. Plasticizer content has a significant effect on changing the mechanical properties of PU. PUs that contain less plasticizer have consistent high tensile stress, tensile modulus, flexural stress, flexural modulus, hardness, Izod impact energy density, stiffness, and glass transition temperature with low failure strains. The elongation capacities increased with increasing plasticizer content, while the brittleness of PU was reduced. The strain energy modulus values obtained by the tensile test showed a decrease in resilience modulus from PU0 to PU15. The optimum toughness modulus was found in PU6. Results show a higher ratio of 10 to 70 between the resilience and toughness moduli. This finding implies that PU can absorb a significant amount of energy as strain energy even after yielding. The findings of this study suggest that palm-based PU elastomer may be applied as a protective coating material for strengthening concrete structures under blast and impact loadings. To control damage sustained by the reduction of the crushing and fragmentation of reinforced concrete structures, PU2–PU8 have the potential to be used as strengthening material in concrete considering their overall behavior, high strain characteristics, and reasonable moduli and strength properties.

## Figures and Tables

**Table 1:** Mechanical and physical properties of selected elastomeric polymers (PU and polyurea) in the quasi-static range.

Parameters	Davidson *et al*. (2004) [9]	Bahei-El-Din and Dvorak (2007a; 2007b) [29,30]		Tekalur *et al*. (2008) [33]	Shim & Mohr (2009) [64]	Raman *et al*. (2011) [37]	Yi *et al*. (2006) [51]	Sayed *et al*. (2009) [19]	Grujicic *et al*. (2010, 2012b) [35,36]	Mohotti *et al*. (2014) [25]
Polymer type	Polyurea	Polyurea	PU	Polyurea	Polyurea	Polyurea	PU	Polyurea	PU	Polyurea
Modulus of elasticity, E (MPa)	234	2,520	1,500	11.16	100	49.5	–	–	689	–
Tangent modulus, E_tan_ (MPa)	23	11	10	–	–	1.9	–	–	–	–
Yield stress (MPa)	11	11	10	–	–	5.5	–	–	–	–
Strain at rupture	0.89	–	–	3.5	–	–	–	–	–	–
Stress at rupture, σ (MPa)	14	–	–	–	–	–	–	–	–	–
Poisson‘s ratio, ν	0.4	0.465	0.463	–	0.448	–	–	0.495	–	–
Modulus of rigidity, G (MPa)	83.6	860	513	–	34.5	–	–	–	–	–
Bulk modulus, k (MPa)	390	–	–	–	320.5	–	–	–	–	–
Tensile strength (MPa)	14	–	–	20.34	–	10.7	–	–	62	–
Mass density, ρ (MPa)	1,442	1,070	1,200	–	1,000	950	1,100	1,070	1,140	1,065
Flexural Strength (MPa)	–	–	–	–	–	–	–	–	89	–
Flexural Modulus (MPa)	–	–	–	–	–	–	–	–	2,020	–

**Table 2:** Glass transition temperature of 8 types of PU (PU0–PU15 are types of PU and PU6—PU which contain 6% PEG).

PU Type	*T*_g_ (°C)
PU0	79.7
PU2	68.9
PU4	59.6
PU6	59.0
PU8	58.5
PU10	56.2
PU12	55.0
PU15	52.7

## Abbreviations

The following abbreviations are used in this manuscript:

ATR: Attenuated total reflectance
Da: Dalton
DSC: Differential scanning calorimetry
$\frac{dq}{dt}$: Change in enthalpy
$\frac{dH}{dt}$: Heat flow
FT-IR: Fourier transform infrared
MDI: 4,4-diphenylmethane diisocyanate
*M*_w_: Molecular weight
PKO-p: Palm kernel oil-based monoester polyol
PEG: Polyethylene glycol
PU: Polyurethane
PU0, PU2, PU4, PU6, PU8, PU10, PU12, and PU15: PU8 indicates the PU that contained 8% *w*/*w* of PEG
*T*_g_: Glass transition temperature
*U*_r_: modulus of resilience
*U*_t_: modulus of toughness
*w*/*w*: Weight/Weight
